# 4-methylumbelliferone-mediated polarization of M1 macrophages correlate with decreased hepatocellular carcinoma aggressiveness in mice

**DOI:** 10.1038/s41598-021-85491-0

**Published:** 2021-03-18

**Authors:** Marcelo M. Rodríguez, Agostina Onorato, María José Cantero, Luciana Domínguez, Juan Bayo, Esteban Fiore, Mariana García, Catalina Atorrasagasti, Ali Canbay, Mariana Malvicini, Guillermo D. Mazzolini

**Affiliations:** 1 Gene Therapy Laboratory, Facultad de Ciencias Biomédicas, Instituto de Investigaciones en Medicina Traslacional, CONICET-Universidad Austral, Av. Presidente Perón 1500 (B1629ODT) Derqui-Pilar, Buenos Aires, Argentina; 2 Department of Medicine, Universitätsklinikum Knappschaftskrankenhaus Bochum, Ruhr-Universität Bochum, Bochum, Germany; 3 Liver Unit, Hospital Universitario Austral, Universidad Austral, Buenos Aires, Argentina

**Keywords:** Liver cancer, Hepatocellular carcinoma, Cancer microenvironment

## Abstract

Hepatocellular carcinoma (HCC) arises in the setting of advanced liver fibrosis, a dynamic and complex inflammatory disease. The tumor microenvironment (TME) is a mixture of cellular components including cancer cells, cancer stem cells (CSCs), tumor-associated macrophages (TAM), and dendritic cells (DCs), which might drive to tumor progression and resistance to therapies. In this work, we study the effects of 4-methylumbelliferone (4Mu) on TME and how this change could be exploited to promote a potent immune response against HCC. First, we observed that 4Mu therapy induced a switch of hepatic macrophages (Mϕ) towards an M1 type profile, and HCC cells (Hepa129 cells) exposed to conditioned medium (CM) derived from Mϕ treated with 4Mu showed reduced expression of several CSCs markers and aggressiveness. HCC cells incubated with CM derived from Mϕ treated with 4Mu grew in immunosuppressed mice while presented delayed tumor progression in immunocompetent mice. HCC cells treated with 4Mu were more susceptible to phagocytosis by DCs, and when DCs were pulsed with HCC cells previously treated with 4Mu displayed a potent antitumoral effect in therapeutic vaccination protocols. In conclusion, 4Mu has the ability to modulate TME into a less hostile milieu and to potentiate immunotherapeutic strategies against HCC.

## Introduction

Hepatocellular carcinoma (HCC) is the 4th cause of cancer-related death worldwide, and its incidence and mortality are increasing steadily^1^. HCC is also the most common cause of death in patients with cirrhosis^2^. Curative therapies are available for a minority of patients with HCC (~ 40%), and although new systemic drugs have been approved for advanced disease (sorafenib and lenvatinib in first line, and regorafenib, cabozantinib and ramucirumab in second-line post sorafenib) their impact on patient survival remain modest^3–5^. Several reports demonstrated that HCC is considered an immunogenic tumor^6,7^, and therefore susceptible for immunotherapy-based strategies such as adoptive T cell therapy using tumor infiltrating lymphocytes or peripheral blood mononuclear cells, therapeutic vaccination with dendritic cells (DCs), systemic cytokines, and more recently immune checkpoint inhibitors (ICIs)^8^. The CTLA-4 and PD-1/PD-L1 ICIs have demonstrated efficacy in patients with HCC^9,10^. Based on a potent overall response rate and duration of response, the combination of nivolumab and ipilimumab was recently approved for HCC patients previously treated with sorafenib^11^. However, the suppressive HCC microenvironment is still a major obstacle for an effective antitumor response particularly for immunotherapeutic strategies^12,13^. The tumor microenvironment (TME) is composed by a complex network of tumor cells and stroma components all mixed in an altered extracellular matrix (ECM). TME components include immune cells, cancer-associated fibroblasts (CAFs), tumor-associated macrophages (TAMs) and cancer stem cells (CSCs), among others^14^. Cellular and non-cellular components of the TME constitute a permissive niche for HCC growth and dissemination^12,14^. It has been reported that type 2 polarized (M2) TAMs suppress antitumor immunity, and the level of infiltrating TAM has been correlated with a poor prognosis^15^. In addition, M2 TAMs release epidermal growth factor (EGF) and transforming growth factor beta (TGF-β), chemokines, metalloproteinases and other cytokines^16^. M2 TAMs are also involved in ECM remodeling, angiogenesis, epithelial-mesenchymal transition (EMT), invasion and metastasis^17^. Furthermore, M2 TAMs interact with other immune cells such as effector lymphocytes promoting resistance against targeted and immunotherapy-based therapies^18,19^.

Tumor-initiating or CSCs are a small population of TME cells intimately related to resistance to therapies^20^. CSCs are characterized by the presence of specific markers including CD133, CD44, CD90, EpCAM, CD13, CD47, SOX-2, Wnt, Oct4, among others^21^, which are involved in their capacity to initiate tumor growth, to form colonies, and to evade the antitumor immune response^22^. In particular, CD133 is a highly recognized marker for CSCs in HCC, and it has been demonstrated that CD133^+^ HCC cells have a potent in vivo tumorigenic capacity^23^. It has been observed that TAMs promote HCC development, and support the expansion of CSCs by IL-6 secretion, highlighting the complex interaction between the components of the TME to sustain tumor growth^24^.

We have previously reported that the coumarin 4-methylumbelliferone (4Mu) selectively inhibits the synthesis of hyaluronan, a principal component of ECM, exerting antitumor effect against HCC in mice^25^. In this work, we wanted to study the effects of 4Mu on the phenotype and composition of hepatic TAMs, and to analyze the cross-talk between hepatic TAMs and CSCs, and to assess how this molecule is able to modulate the TME milieu to increase the action of the immune system.

## Results

### In vivo 4Mu therapy induces hepatic macrophages polarization towards a M1 profile

**Figure 1:**
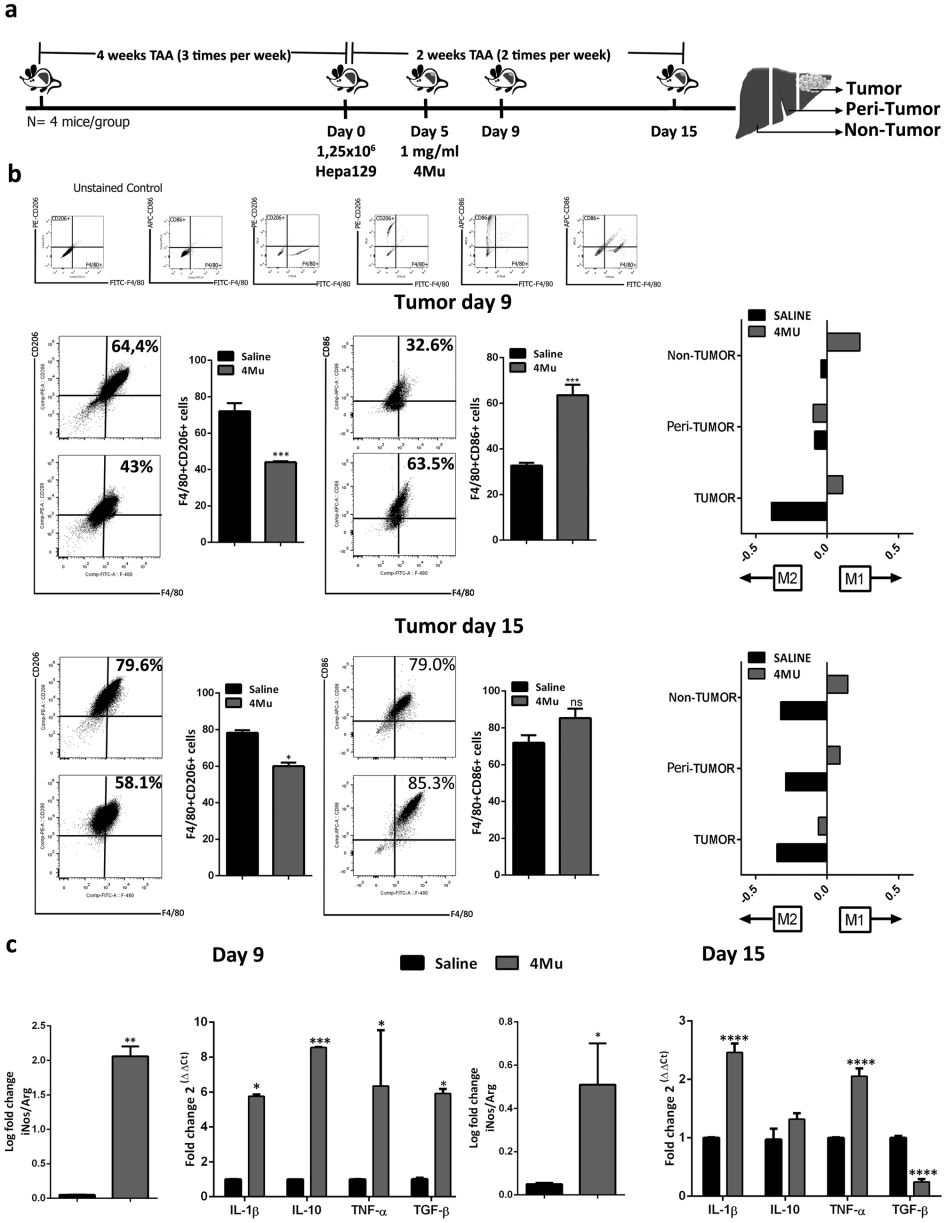
In vivo modulation of hepatic macrophages expression profile by 4Mu. (**a**) *Left* in vivo experimental model (6 to 8-week-old male C3Hj/He mice; n = 8/group were injected with TAA (200 mg/kg; i.p.) for 4 weeks, 3 times per week, to induce fibrosis. Then, all mice received an intrahepatic inoculation of 1.25 × 10^5^ syngeneic Hepa129 cells (day 0). After tumor implantation, mice received 200 mg/kg 4Mu in drinking water (day 5). On day 9 (n = 4/group) and day 15 (n = 4/group) after 4Mu initiation, mice were sacrificed and liver samples (n = 4/group) were collected. *Right* The livers were perfused with collagenase and separated in 3 sections (peri-tumoral, tumoral, and non-tumoral tissue). Isolation of non-parenchymal cells from each tissue section was carried out. (**b**) Representative dot plots of flow cytometry analysis using BD Accuri C6 propietary software version 1.0.264.21 (www.AccuriCytometers.com) of auto fluorescence (upper left panels), F4/80^+^, CD206^+^ and CD86^+^ on non-parenchymal cells. Bar graphs showed the percentage of CD206^+^ F4/80^+^ (M2), and CD86^+^ F4/80^+^ (M1) cells in tumor tissue on day 9 (***p < 0.001) and day 15 (*p < 0.05); 4Mu vs. saline, Mann–Whitney test. Mϕ type1/type2 proportion was calculated as log10 (CD86^+^/CD206^+^). (**c**) mRNA expression levels of iNOS, Arg1, IL-1β, IL-10, TNF-α, and TGF-β on isolated Mϕ from tumor tissue samples. iNOS /Arg1 ratio **p < 0.01 and *p < 0.05 4Mu vs. Saline (day 9 and day 15 respectively); Mann–Whitney test; TGF-β, IL-1β and TNF-α *p < 0.05; IL-10 ***p < 0.005; 4Mu vs. saline (day 9); TGF-β, IL-1β and TNF-α ****p < 0.001; 4Mu vs. saline (day 15); two-way ANOVA. Data are expressed as the mean ± SEM. The experiment was carried-out 2 times.

Macrophages (Mϕ) are major components of TME, and they have a pivotal role in promoting HCC progression^26^. We have studied the effects of 4Mu on hepatic Mϕ population in our experimental model of HCC with associated-fibrosis^25^. After 4 weeks of TAA administration, mice were inoculated orthotopically with 1.25 × 10^5^ Hepa 129 cells (day 0). On day 5, animals received daily saline or 4Mu orally (200 mg/kg; Fig. 1a, *Left*). On day 9 and 15 mice were sacrificed, and liver sample collected. The fraction of non-parenchymal cells from tumor, peri-tumor, and non-tumor liver regions (Fig. 1a, *Right*) were obtained and analyzed by flow cytometry. The percentage of F4/80^+^CD206^+^ and F4/80^+^CD86^+^ cells was evaluated and the M1/M2 proportion was calculated as log10 (CD86^+^/CD206^+^). 4Mu therapy induced a polarized M1 profile in tumor and non-tumor sections in comparison with saline group (Fig. 1b, *Right*) on day 9. In addition, we observed a M1 Mϕ profile induced by 4Mu in peri-tumor and non-tumor sections on day 15; Fig. 1b also showed the reduced percentage of F4/80^+^CD206^+^ cells (44.0 ± 0.63% vs. 72.0 ± 4.41%; ***p < 0.001 and 60.0 ± 1.90% vs. 78.2 ± 1.43%, *p < 0.05 4Mu vs. saline, respectively. This effect is accompanied by an increase in the percentage of F4/80CD86^+^ cells in tumor pieces (63.5 ± 4.63% vs. 32.6 ± 1.24%; *p < 0.05 and 85.2 ± 5.22% vs. 71.8 ± 4.12%; *p < 0.05 4Mu vs. saline) induced by 4Mu on day 9 and day 15, respectively. We also found that the ratio of mRNA levels of iNOS/Arg1 in hepatic Mϕ isolated from tumor sections of 4Mu treated mice was enhanced both at day 9 and at day 15 (**p < 0. 01 on day 9; *p < 0.05 on day 15; Fig. 1c). Although the levels of cytokines modulated in tumor hepatic Mϕ on day 9 did not show clearly a definitive profile, there was a significant increase in the mRNA levels of IL1-B and TNF-α together with a decrease of TGF-β and IL-10 on day 15, which means a clear polarization induced in tumor hepatic M towards an M1 profile after 4Mu treatment (Fig. 1c).

### 4Mu modifies the macrophages profile towards a pro-inflammatory phenotype

**Figure 2:**
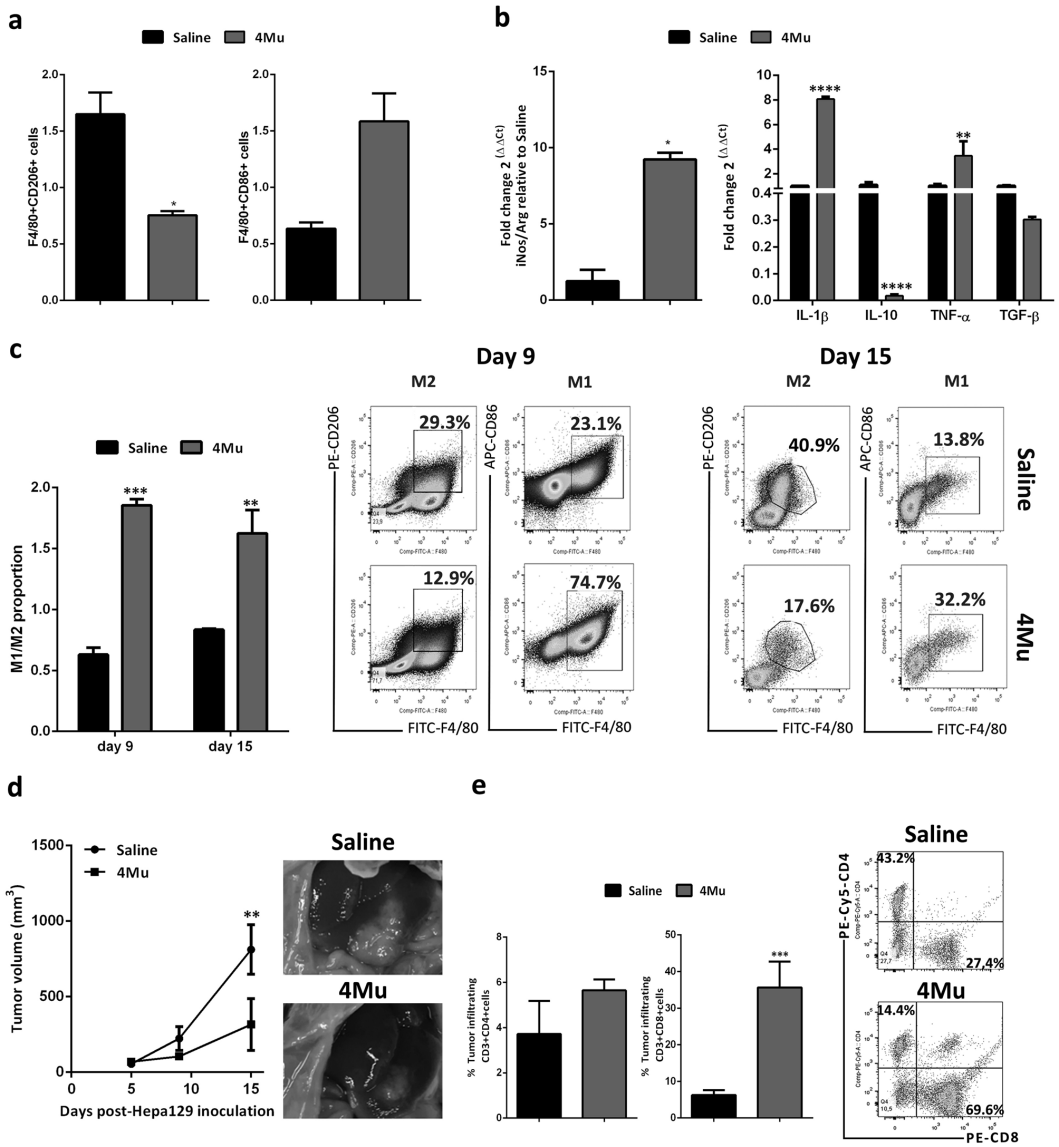
Type 1 macrophages phenotype was induced by 4Mu. (**a**) Peritoneal macrophages (pMϕ) were isolated from healthy mice (n = 4), cultured with 0.5 mM 4Mu for 72 h (n = 2), stained with anti-F4/80, anti-CD86 and anti-CD206 antibodies, and analyzed by flow cytometry. (**b**) iNOS/Arg1 ratio, and cytokine gene expression were measured in pMϕ by qPCR (*p < 0.05; **p < 0.01; ****p < 0.0001 4Mu vs. saline; Mann–Whitney test and two-way ANOVA test respectively). (**c**) In vivo effects of 4Mu on pMϕ in mice (n = 4/group) with fibrosis-associated HCC. pMϕ were obtained on day 9 (n = 2) and 15 (n = 2). Flow cytometry of F4/80^+^ CD86^+^ and CD206^+^ cells were analyzed, and the M1/M2 proportions were calculated as log2 (CD86^+^/CD206^+^);***p < 0.001, **p < 0.01 4Mu vs. saline, on days 9 and 15, respectively; Mann–Whitney test. (**d**) Tumor volume was measured on 4Mu treated and non-treated tumor-bearing mice at day 9 (p = 0.0571; ns) and at day 15 (**p < 0.01; Unpaired T Test). (**e**) Lymphocytic infiltration of HCC tumors. Flow cytometry analysis of CD4 and CD8 T cells. Percentage of CD8^+^ cells ***p < 0.001 4Mu vs. saline, Mann–Whitney Test. Data are expressed as the mean ± SEM. Flow cytometry was analyzed using BD Accuri C6 propietary software version 1.0.264.21 (www.AccuriCytometers.com) The experiments were repeated 2 times.

Under conditions of an immunosuppressive TME, stroma cell-derived factor 1 (SDF-1), secreted by activated hepatic stellate cells (HSCs) and tumor-derived vascular endothelium growth factor (VEGF) regulate TAMs recruitment and induce their polarization toward an M2 profile^14^. We have previously reported that 4Mu decreased the activation of HSCs leading to a reduction in the degree of liver fibrosis in mice^27^, and a decrease in the production of VEGF, SDF-1 and IL-6^28^. Then, we wonder whether the hepatic Mϕ profile generated upon 4Mu therapy might be due to a direct effect on hepatic Mϕ. To elucidate this, we in vitro cultured isolated peritoneal Mϕ (pMϕ) from healthy micewith 4Mu. After 72 h, we tested F4/80^+^CD86^+^ and F4/80^+^CD206^+^ cells by flow cytometry, and measured mRNA levels of M1 and M2 cytokines. We observed that 4Mu reduced the percentage of F4/80^+^CD206^+^ in comparison with control (12.1 ± 0.81 vs. 25.0 ± 4.42, *p < 0.05, day 9; and 17.6 ± 2.83 vs. 39.3 ± 1.61, *p < 0.05, day 15, Mann–Whitney test) (Fig. 2a). We observed that pMϕ treated in vitro with 4Mu showed a fivefold increase in the ratio iNOS/Arg1 (*p < 0.05, 4Mu vs. RPMI; Mann–Whitney test). In addition, the mRNA levels of pro-inflammatory cytokines were significantly increased (****p < 0.001 and ***p < 0.005 respectively; 4Mu vs. RPMI, two-way ANOVA test) while mRNA levels of IL-10, understanding that pMϕ from male mice can express high levels of IL-10^29^, were reduced (****p < 0.001; Fig. 2b). These results suggest a direct effect elicited by 4Mu on Mϕ profile.

Then, we assessed whether the effect of 4Mu on hepatic Mϕ polarization could be monitored by studying pMϕ. Mice with fibrosis-associated HCC were treated or not with 4Mu, and pMϕ were obtained at days 9 and 15. 4Mu treatment induces an increase in M1/M2 profile both at day 9 and 15 after therapy (Fig. 2c, *Left*). The dot plot graph showed the representative percentage of F4/80^+^CD206^+^ and F/480^+^CD86^+^ cells on pMϕ obtained from treated HCC-bearing mice (Fig. 2c*, Right*). To validate the antitumor effect induced by 4Mu in our model, mice were sacrificed, and tumor growth was analyzed. Figure 2d showed the efficacy of 200 mg/kg 4Mu on orthotopic HCC tumor nodules. Finally, we assessed the percentage of tumor infiltrating T cells, as they play a key role after M1 polarization induced by 4Mu. Figure 2e illustrated that 4Mu improved the percentage of CD3^+^CD4^+^ (5.64 ± 0.48% vs. 3.71 ± 1.43%; ns, 4Mu vs. Saline) and CD3^+^CD8^+^ T cells (35.6 ± 7.01% vs. 6.25 ± 1.34%; (***p < 0.001, 4Mu vs. Saline; Mann–Whitney Test) in tumor tissue sections from treated mice in comparison with non-treated mice We also observed that 4Mu therapy was able to induce a significant decrease of splenic Gr1^+^CD11b^+^ myeloid derived suppressor cells (MDSCs), and CD4^+^Foxp3^+^ Regulatory T cells (Tregs) levels in HCC-bearing mice (Supplementary Fig. S2).

### Effect of M1 macrophages polarization induced by 4Mu on hepatocarcinogenesis

**Figure 3:**
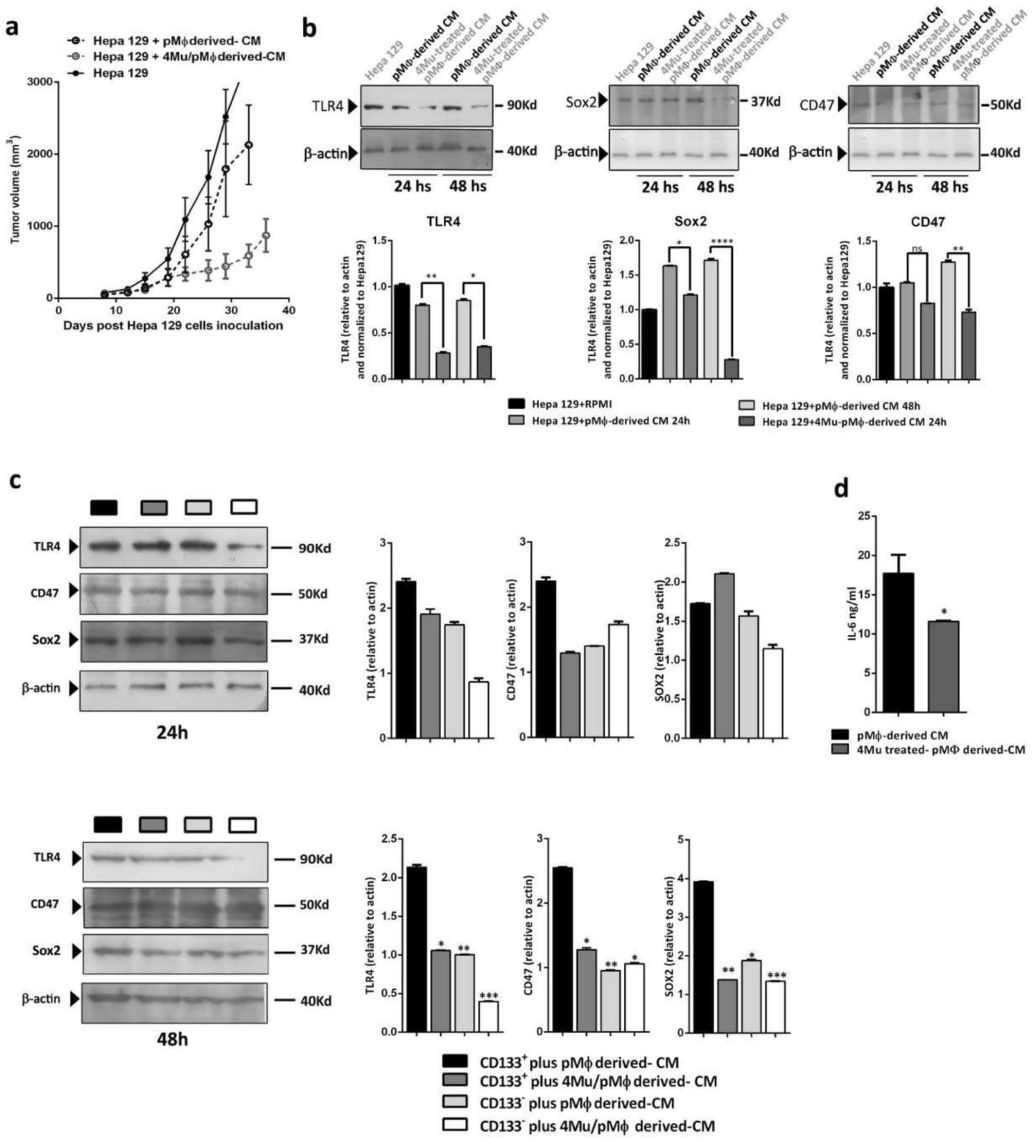
M1 type macrophages induced by 4Mu ameliorate hepatocellular carcinoma aggressiveness. (**a**) Hepa129 cells were incubated for duplicate with RPMI (Hepa129 + RPMI), CM from isolated pMϕ (Hepa129 + pMϕ-derived CM), and CM from isolated pMϕ in vitro treated with 4Mu (Hepa129 + 4Mu-treated pMϕ-derived CM) for 48 h. Pre-conditioned 1 × 10^6^ Hepa 129 cells were injected s.c. in C3Hj mice (n = 4/group), and tumor volume was measured. (**b**) Hepa129 cells were cultured with RPMI, pMϕ-derived CM and 4Mu-treated pMϕ-derived CM, and the expression levels of TLR4, CD47 and Sox2 were analyze by western blot; different parts from different membranes were delineated with dividing lines. Full-length blots are presented in Supplementary Fig. 4A **p < 0.05, *p < 0.01 or ****p < 0.001; Kruskal-Wallys test. (**c**) TLR4, CD47 and Sox2 expression was also determined by western blot on magnetic-isolated CD133^+^ and CD133^-^ Hepa129 cells cultured with pMϕ-derived CM or 4Mu-treated pMϕ-derived-CM; different parts from different membranes were delineated with dividing lines. Full-length blots are presented in Supplementary Fig. S4B, **p < 0.01CD133 + plus 4Mu-treated pMϕ-derived-CM vs. pMϕ-derived CM; 48hs Kruskal–Wallys test. (**d**) IL-6 production by pMϕ treated or not with 4Mu in vitro by ELISA, *p < 0.05 Unpaired T test; Data are expressed as the mean ± SEM. The in vivo and in vitro experiments were carried-out 2 times.

We next aimed to evaluate if the M1 phenotype of Mϕ induced by 4Mu has effects on the capability of HCC cells to establish and growth. To this end, s.c. tumors were developed in C3H/He mice with Hepa129 cells cultured in the presence of CM derived from pMϕ treated or not with 4Mu (4Mu-treated pMϕ derived CM). Remarkably, we found a high index of tumor development and progression in Hepa129 control tumors generated by cells incubated with either RPMI or with CM derived from untreated pMϕ. On the other hand, the growth of tumors established from Hepa129 cells pre-treated with 4Mu-treated pMϕ derived-CM was significantly reduced (Fig. 3a) Then, we wanted to study if tumor aggressiveness was modified by 4Mu-treated pMϕ derived CM and untreated pMϕ-derived CM). We observed that after 24 h and 48 h of pre-conditioning, 4Mu-treated pMϕ derived-CM reduced the expression levels of cancer stemness markers (TLR4 and CD47)^30,31^, and the totipotency factor Sox2^32^ in cells (*p < 0.05, **p < 0,01 and ****p < 0,001; Kruskal–Wallis test) (Fig. 3b). As we described, HCC are composed of subpopulations of tumor cells with diverse tumorigenic abilities^33^. Then, we magnetically isolated CD133^+^ and CD133^-^ Hepa129 cells to compare if CM mediate a differential effect mainly on CSCs. Figure 3c showed that 24 h of pre-conditioning with 4Mu treated-pMϕ derived-CM has no effect on the CSCs markers levels while 48 h of pre-conditioning with 4Mu treated-pMϕ derived-CM strongly reduces levels of TLR4, CD47 and Sox2 expression on CSCs. In addition, CM derived from 4Mu-treated pMϕ showed a reduced amount of IL-6 in comparison with untreated pMϕ (Fig. 3d).

**Figure 4:**
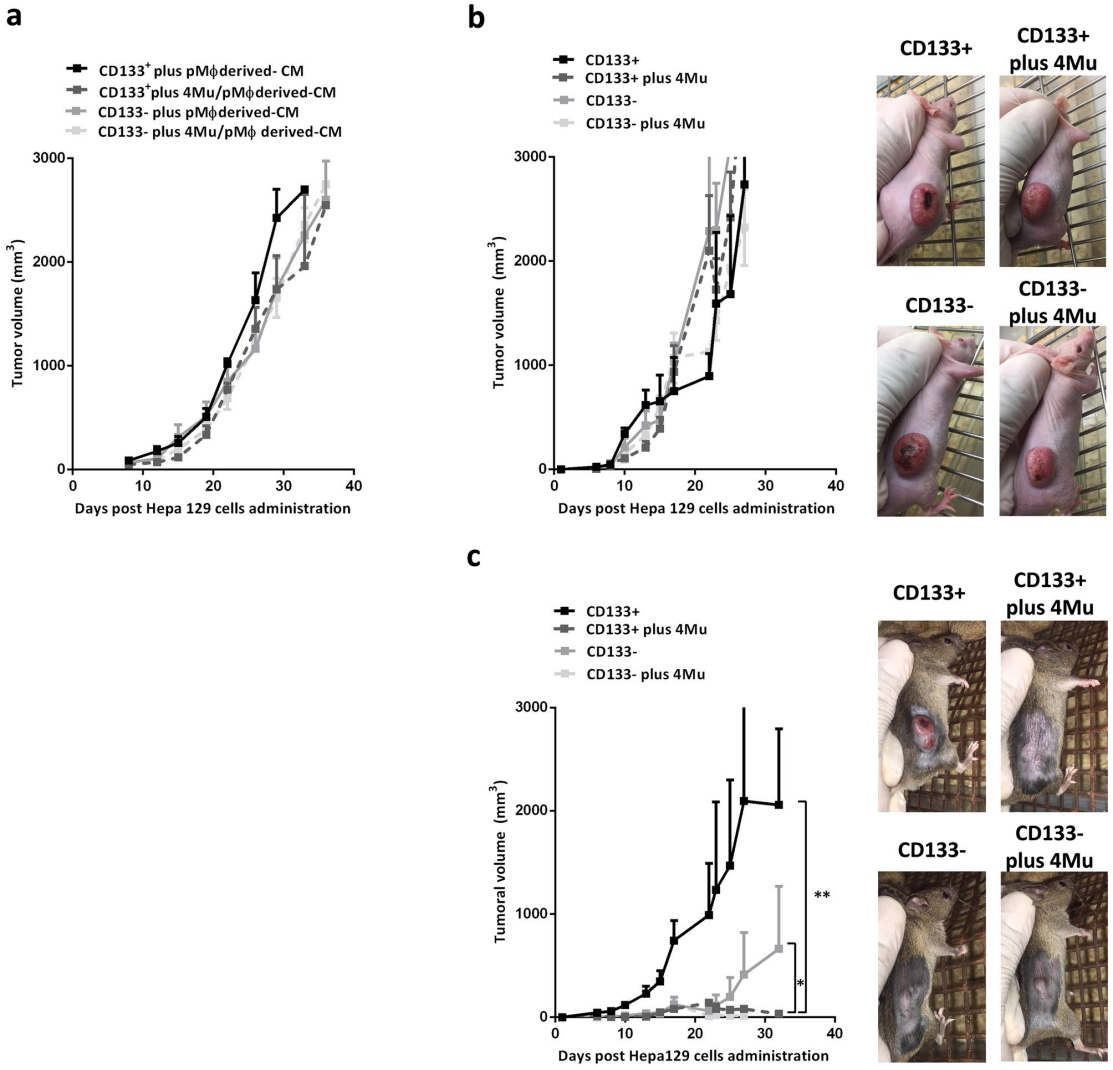
4Mu and M1 hepatic macrophages facilitate immune recognition of CD133^+^ HCC cells, and promote antitumor response. (**a**) Hepa129 cells were incubated for duplicate with RPMI (Hepa129 + RPMI), CM from isolated pMϕ (Hepa129 + pMϕ-derived CM) and CM from isolated pMϕ in vitro treated with 4Mu (Hepa129 + 4Mu-treated pMϕ-derived CM) for 48 h. Pre-conditioned 1 × 10^6^ Hepa129 cells were injected s.c. in Nu/Nu mice (n = 4/group), and tumor volume was measured. (**b**) Nu/Nu mice (n = 4) and (**c**) immunocompetent C3Hj mice (n = 4) were injected s.c. with 1 × 10^5^ CD133 + Hepa129 cells, 1 × 10^5^ 4Mu-treated CD133^+^ Hepa129 cells, 1 × 10^5^ CD133-Hepa129 cells, and 1 × 10^5^ 4Mu-treated CD133- Hepa129 cells. Tumor volume was monitored by caliper. **p < 0.01 CD133+ vs. 4Mu-treated CD133+; *p < 0.05 CD133- vs. 4Mu-treated CD133+; Tukey’s multiple comparisons test. Data are expressed as the mean ± SEM. The experiment was carried-out 2 times.

We next administrated Hepa 129 cells exposed to CM from 4Mu treated-pMϕ in athymic Nu/Nu mice. The results showed that tumor progression in immunosuppressed mice with Hepa129 cells pretreated with pMϕ derived-CM or 4Mu treated-pMϕ derived-CM were similar to controls (Fig. 4a).

We previously demonstrated that 4Mu does not show a significant impact on Hepa129 cell growth and survival. However, 4Mu can modulate the expression of CSCs markers, particularly CD47, contributing to the phagocytosis of CD133^+^ CSCs^25^. Based on this, modulation of CD47 expression might have a dual effect, on one side induces a less aggressive tumor phenotype and, on the other side, HCC cells might become more susceptible to the recognition by the immune system. Figure 4b,c reveal that CD133^+^ cells could generate tumors in both immunocompetent and immunocompromised mice. In contrast, when the expression of CD47 is inhibited by 4Mu, tumors progressed only in immunocompromised animals while were potently controlled in immunocompetent mice.

### 4Mu stimulates antigen presentation by dendritic cells and improves their capability to phagocyte cancer cells

**Figure 5:**
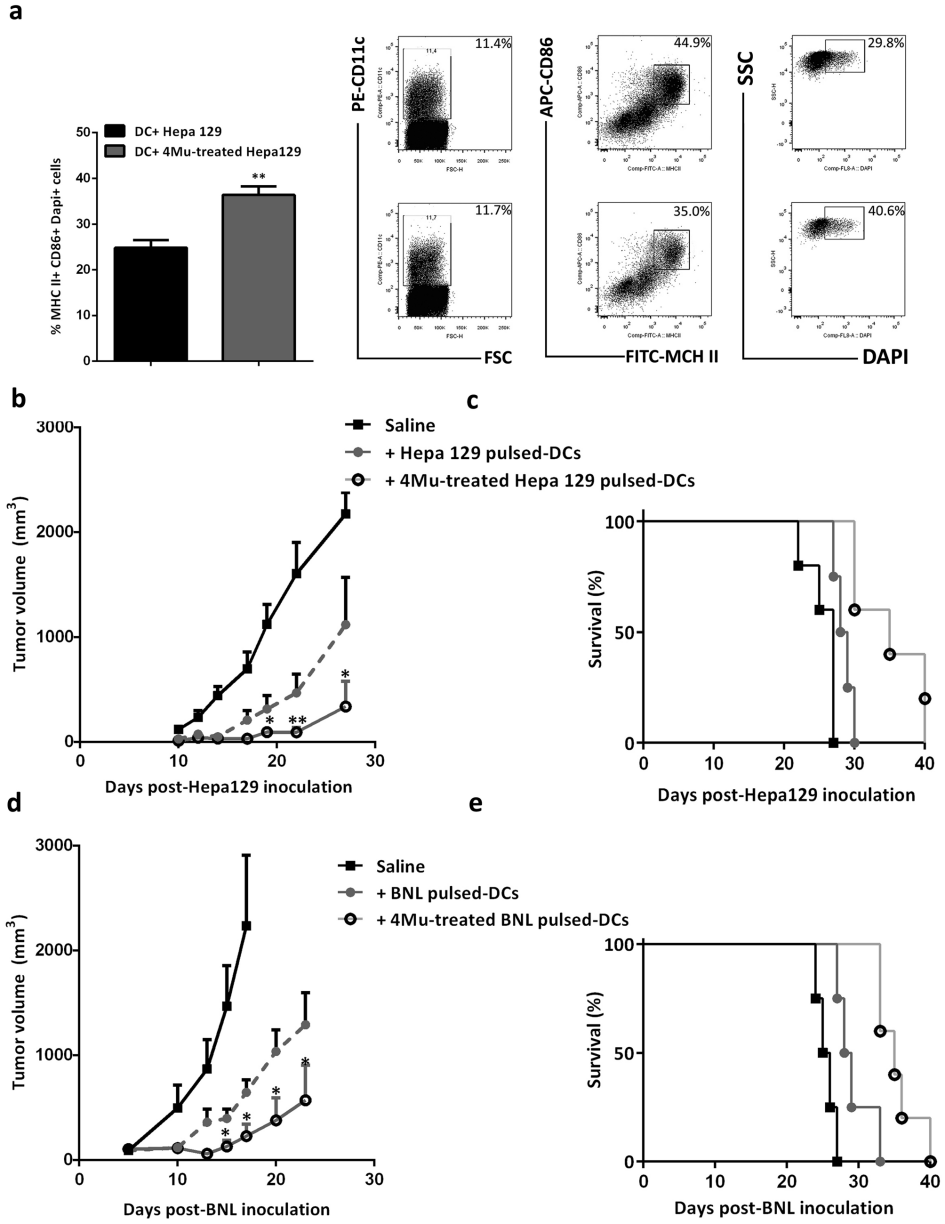
4Mu preconditioning of HCC cells increases phagocytosis of Hepa129 cells by DCs promotes a potent antitumoral response. (**a**) DCs was derived from bone marrow of C3H/j mice, and incubated for 7 days in presence of GM-CSF and IL-4. Hepa129 cells were treated with 4Mu for 72 h. Phagocytosis assay of DCs was performed by incubation with DAPI + Hepa129 cell (1:4). The analysis of the percentage of MHCII^+^CD86^+^DAPI^+^ (right) was performed using flow cytometry *p < 0.05 4Mu vs. RPMI (left), Mann–Whitney test. Data are expressed as mean percentage ± SD. (**b**) C3Hj mice were s.c. inoculated with 1 × 10^6^ Hepa 129 cells (n = 4–5/group); on day 9, mice were peritumorally injected with 1 × 10^6^ 4Mu-treated tumor-pulsed DCs, tumor-pulsed DCs o saline *p < 0.05 4Mu-treated Hepa 129-pulsed DCs vs. Saline; (**c**) Kaplan–Meier survival curve of the mice bearing Hepa 129 tumors. (**d**) BALB/c mice (n = 4/group) were s.c. inoculated with 1 × 10^6^ BNL cells; on day 9, mice were peritumorally injected with 1 × 10^6^ 4Mu-treated BNL- pulsed DCs, BNL-pulsed DCs o saline. *p < 0.05 4Mu-treated BNL-pulsed DCs vs. Saline; ANOVA and Tukey’s test. (**e**) Kaplan–Meier survival curve of the mice bearing BNL tumors. Tumor volumes were measured 3 times a week over a period of 30 days. The growth curve is representative of 3 independent experiments (mean ± SD).

We assessed whether 4Mu have effects on the function of dendritic cells (DCs). First, we performed an in vitro phagocytosis assay using BM-derived DCs from C3H/He mice. Hepa129 HCC cells were labeled with DAPI, co-cultured with DCs for 2 h, and incubated with MHC II and CD86 antibodies. We quantified the presence of MHC II^+^ CD86^+^ DCs, gated them, and then identified MHCII^+^CD86^+^DAPI^+^ phagocyted Hepa 129 cells (Fig. 5a, *Right*). Interestingly, phagocytosis was significantly increased in Hepa129 + 4Mu cells compared with untreated Hepa129 cells (Fig. 5a, *Left*; 36.4 ± 3.69 vs. 24.8 ± 3.40; *p < 0.05, Mann–Whitney test). We also observed that 4Mu could facilitate the maturation of DCs since the percentages of CD11^+^MHCII^+^CD86^+^ DCs were higher when DCs were exposed to 4Mu for 72 h (Supplementary Fig. S3). This result suggests that the ability to recognize tumor cells by DCs is increased by 4Mu.

### Therapeutic vaccination using 4Mu treated-tumor-pulsed dendritic cells delayed HCC growth

We studied the effect of a therapeutic vaccine generated with DCs pulsed with 4Mu treated Hepa129 whole tumor lysates. Then, we injected DCs pulsed with Hepa129 tumor lysates obtained from 4Mu- or vehicle-treated mice (DCs/Hepa129/4Mu or DCs/Hepa129 respectively; i.p.) in C3H/He tumor-bearing mice. Figure 5b showed that vaccination with DCs/Hepa129/4Mu induced a potent antitumoral effect in comparison with DCs/Hepa129 (*p < 0.05; Kruskal–Wallis test). Additionally, DCs/Hepa129/4Mu therapy significantly increased the animal survival when compared to control mice (Fig. 5c; Log-rank test *p < 0.01). To validate these results, we also evaluated the activity of DCs vaccination in a murine cholangiocarcinoma (BNL cells) model. In this case, mice that received DCs pulsed with tumors treated with 4Mu also showed a significant decrease in tumor growth and an increase in animals survival (Fig. 5d,e; *p < 0.05, Kruskal–Wallis test; *p < 0.01, Log-rank test respectively).

## Discussion

In the last years, the study of the TME components on carcinogenesis has gained an special interest due their potential as therapeutic targets^34^. Different cellular components of the TME could act in synergy to facilitate cancer progression and to avoid the immune system recognition. In particular, TAMs population and their activation status are involved in tumor aggressiveess^15^. The classical Mϕ activation M1 type is characterized by the expression of the co-stimulatory molecule CD86, an increase in the activity of iNOS, and in the production of pro-inflammatory cytokines such as IL-1β and TNF-α. On the other hand, the alternative type 2 polarized Mϕ (M2) is known by the expression of CD206, an increased activity of arginase I, and by the release of the anti-inflammatory cytokines IL-10 and TGF-β^17^.

Hepatic Mϕ play an essential role in the pathogenesis of chronic inflammatory liver diseases, and in the progression of HCC^26,35^. TAMs are one of the more abundant immune cells that infiltrate tumors. Yeung et al*.* described a direct effect M2 Mϕ on HCC growth. They showed that the expansion of TAMs with M2 phenotype promote tumor growth and invasiveness through the release of CCL22. Moreover, the peritumoral accumulation of M2 Mϕ observed in patient samples correlates with poor prognosis, and constitutes a predictor of patient survival^15^.

The role of circulating stem cell-like tumor cells phenotypes in HCC was addressed by Sun et al. They observed that the presence of circulating HCC cells with a stem-like phenotype (Epcam^+^/CD133^+^) was associated with recurrence in HCC patients after surgery^36^. In addition to CCL22, it has been demonstrated that M2 TAMs also secrete TGF-β and IL-6 promoting EMT and acquisition of CSCs-like properties^24,37^. IL-6 detected in human HCC samples correlated with the presence of CSCs markers, and IL-6 expressed by TAMs induces the expansion of CD44^+^ cells in culture^24^.

Our previous results showed that 4Mu mitigates thioacetamide-induced liver chronic injury by reducing hyaluronan deposition, and hepatic stellate cells activation. 4Mu therapy also inhibited angiogenesis and tumor growth in vivo^28,38^. In the present work, we demonstrated that 4Mu modified the hepatic Mϕ phenotype. and their cytokine secretion profile. While a typical M2 activation was found in Mϕ from tumor, peri-tumor, and non-tumor tissues of untreated HCC, there was a significant M1 polarization induction in tumoral Mϕ after 4Mu treatment. When HCC cells were cultured in presence of supernatant from 4Mu-induced M1 Mϕ, and challenge immunocompetent mice with this preconditioned HCC cells, a delayed tumor progression was observed. This data suggests that 4Mu modulates the ability of hepatic Mϕ to promote tumor growth. Remarkably, CM derived from Mϕ treated with 4Mu induces a significant reduction of stemness-related markers on both whole HCC cells and more significant on isolated CSCs.

Recently, it has been reported that increased expression of CD47 on HCC cells was positively correlated with the density of hepatic Mϕ, and with poor clinical prognosis^39^. In this work, the authors also suggested that IL-6 derived from hepatic TAMs was involved in the up regulation of CD47 expression on HCC cells. In this line, we observed in our model that CM derived from Mϕ treated with 4Mu showed lower levels of IL-6, and that 4Mu reduced the stemness-related phenotype on HCC cells and their capacity to growth in vivo after the exposure to CM derived from Mϕ treated with 4Mu.

Conditioned media from Mϕ treated with 4Mu induced a less aggressive HCC phenotype and facilitated immune system recognition. We also demonstrated that CD133^+^ CSCs generate tumors in both immunocompetent and immunocompromised mice, but when the expression of stemness-related markers is inhibited by 4Mu, tumors grew only in athymic animals while were susceptible to the immune system control in immunocompetent mice.

It has been proposed that hepatic M2 Mϕ interact with cytotoxic CD8^+^ T cells, and induce resistance to immunotherapy. In addition, a positive association was demonstrated between the expression levels of M2 Mϕ markers, decreased CD8^+^ T cell infiltration, and PD-L1 levels in tumor pieces from patients with HCC^19^. In our hands, M1 polarization was induced in HCC tumors after 4Mu treatment, the percentage of CD3^+^CD8^+^ tumor infiltrating T cells was increased, and by the reduction of CD47 expression on CSCs they might result more “visible” to the immune system, open the possibility to combine 4Mu with other immunotherapies like checkpoints inhibitors^40^.

Drug resistance was reported in advanced HCC patients treated with standard tyrosine kinase inhibitors (TKI)^3,41^. Sorafenib-resistant clones derived from HCC cell lines showed CSCs properties, including up-regulation of CD47. In addition, CD47 expression was found to be regulated by nuclear factor kappa B (NF-κB), and human HCC samples showed a positive correlation between NF-κB and the presence of CD47^42^. Modulation of CSCs markers, particularly CD47, directly by 4Mu or indirectly through the induction of M1 hepatic Mϕ generated by 4Mu could be considered as an approach to sensitize cancer cells to TKI therapy, particularly in patients who have been previously treated with sorafenib.

We showed that 4Mu decreases CD47 expression on HCC cells and facilitates phagocytosis by Mϕ, which is also associated with antitumor immune response in mice. It has been reported a role of SIRP-expressing DC in antitumor responses, including in HCC; it is possible that CD47 down regulation by 4Mu may affect the response mediated by both macrophages and DC. Here, we have also described that 4Mu has the ability to turn DCs more active to recognize and engulf tumor cells. It have been reported that DCs migrate into tumor-draining lymph nodes and prime CD8^+^ or CD4^+^ T cells to induce antitumor responses in mouse models, although their manipulation in cancer vaccination protocols has not reached a potent clinical impact^43,44^. Tumor progression was significantly inhibited in mice that receive DCs pulsed with 4Mu-treated HCC lysate in comparison with mice DCs pulsed with HCC lysate alone, suggesting that 4Mu therapy increases the potential of DCs to generate immunity against HCC. Dendritic cell-based vaccines have been tested as therapeutic tool for HCC^45^. A recently reported meta-analysis aimed to investigate the efficacy of DCs alone or combined with conventional treatments illustrated that cellular immunotherapy improve prognosis by increasing overall survival and reducing recurrence in patients with advanced HCC^46^.

All in all, our results suggest that 4Mu exerts a significant antitumoral effect: (i) by inducing a switch of hepatic Mϕ into a M1 profile, (ii) by reducing their capacity to secrete IL-6, (iii) by increasing HCC recognition by the immune system upon incubation of tumor cells with CM derived from Mϕ treated with 4Mu; (iv) by reducing the expression of several CSCs markers on HCC cells; and (v) by increasing the ability of DCs to inhibit HCC tumor growth in therapeutic vaccination protocols. In conclusion, our data highlight the potential of 4Mu to modulate the TME facilitating the induction of an immune response against HCC.

## Materials and methods

### Animals

Six-to-eight-week-old male C3Hj/He, BALB/c and athymic N:NIH(S)-nu mice (Nu/Nu) mice were purchased from Centro Atómico Ezeiza (Buenos Aires, Argentina). Animals were maintained at our Animal Resources Facilities in accordance with the experimental ethical committee and the NIH guidelines on the ethical use of animals. The Animal Care Committee from School of Biomedical Sciences, Universidad Austral, approved the experimental protocol (protocol #2018-05) which was based on the essential points of the ARRIVE guidelines.

### Cell lines

Hepa129 cells (HCC cells syngeneic with C3H/He mice) were kindly provided by Dr. Volker Schmitz (Bonn University, Germany). BNL cells (cholangiocarcinoma cells, syngeneic with BALB/c mice) were provided by Prof. Dr. Jesús Prieto (CIMA, Spain). Hepa129 and BNL cells were grown in RPMI 1640 (GIBCO-Fisher Scientific, UK) with 10% fetal bovine serum (FBS).

### Drugs

4 Methylumbelliferone (4Mu) sodium salt was purchased from Sigma-Aldrich (USA)^28^.

### In vivo experiments

#### Experimental model of HCC associated with fibrosis

C3H/He mice received 200 mg/kg of thioacetamide (TAA) (Sigma-Aldrich, USA) intraperitoneally (i.p.) 3 times a week for 4 weeks to develop liver fibrosis. TAA-treated livers from tumor-bearing mice showed the extensive appearance of portal–portal and central–portal fibrous septae and distortion of liver architecture (Supplementary Fig. S1). On day 28, mice were anesthetized and orthotopic tumors were established by subcapsular inoculation of 1.25 × 10^5^ Hepa129 cells directly into the left liver lobe by laparotomy^25^ (day 0). Five days after tumor implantation, mice were distributed in groups (n = 8/group) and received: (i) saline (control), or (ii) 4Mu 200 mg/kg in the drinking water ad libitum. TAA was administrated until mice were used in ex vivo studies. On day 9 and 15 mice were sacrificed, tumor volume was measured using a caliper, and liver samples were collected. In some experiments, livers were perfused with collagenase and separated in 3 sections (peri-tumoral, tumoral, and non-tumoral tissue). Isolation of non-parenchymal cells from each tissue section was carried out using Histodenz.

#### Subcutaneous tumor implantation in immunosuppressed and immunocompetent mice

For assessment of the in vivo tumor growth, 6 to eight-week-old male athymic N:NIH(S)-nu mice (Nu/Nu), and C3H/He mice (n = 4/group) were subcutaneously (s.c.) injected into the right flank with 1 × 10^5^ CD133 + Hepa129 cells, 1 × 10^5^ 4Mu-treated CD133 + Hepa129 cells, 1 × 10^5^ CD133-Hepa129 cells and 1 × 10^5^ 4Mu-treated CD133- Hepa129 cells. Tumor volume was determined as above. Also, Nu/Nu and C3H/He mice (n = 4) were s.c. injected with Hepa129 + conditioned medium from peritoneal macrophages (pMϕ derived-CM) and Hepa129 + 4Mu-treated pMϕ derived-CM (described below).

#### Mice vaccination with DCs

HCC tumors were induced s.c. in C3H/He mice (n = 4–5/group) by inoculating 1 × 10^6^ Hepa129 cells, or in BALB/c mice (n = 4/group) by inoculating BNL cells. Tumors were detected approximately 10 days after injection. Then, 1 × 10^5^ matured DCs, or DCs loaded with lysates were injected peritumorally into tumor-bearing mice. Tumor growth was measured 3 times per week, and volumes were calculated as described above.

### Ex vivo experiments

#### Macrophages profile assessment

Histodenz isolated liver cells were cultured for 30 min. Then, cells adhered to plastic were collected, and used for flow cytometry analysis or stored at − 80 °C with Trizol (Invitrogen, USA) for further RNA isolation. Flow cytometry of F4/80+, CD206+ and CD86+ were performed using a FACS Aria (BD Biosciences, USA). For FACS, potential autofluorescence of 4Mu was excluded in all studies and fluorescence minus one controls (FMO) were performed using BD CompBead particles (Cat No 560499). For accurately, gates were placed at the FMO staining. Macrophages type1/type2 proportion was calculated as log10 (CD86^+^/CD206^+^).

#### RNA isolation and quantitative PCR analysis

total RNA samples were isolated using Trizol Reagent (Sigma-Aldrich Co) and Total RNA (2 mg) was reverse transcribed (RT-qPCR) with 200 U of SuperScript II Reverse Transcriptase (Invitrogen) using 500 ng of Oligo (dT) primers. cDNAs were subjected to real time polymerase chain reaction (qPCR) (Stratagene Mx3005p, Stratagene, La Jolla, CA, USA) as we previously described^25^. The mRNA levels of inducible Nitric Oxide Sintetase (iNOS), interleukin-1b (IL-1β), Tumor Necrosis Factor alpha (TNF-α), Arginase I, interleukin-10 (IL-10) and Transforming Growth Factor beta (TGF-β) were quantified using SYBRGreen (Invitrogen); amplifications were carried out using a cycle of 95 °C for 10 min and 40 cycles under the following parameters: 95 °C for 30 s, 56 °C for 30 s, 72 °C for 1 min. At the end of PCR reaction, temperature was increased from 60 to 95 °C at a rate of 2 °C/min, and fluorescence was measured every 15 s to construct the melting curve. Values were normalized to levels of glyceraldehyde-3-phosphate dehydrogenase transcript GAPDH used as housekeeping. The expression of GAPDH was not significantly different between groups. Data were processed by the ΔΔCt method. The relative amount of the PCR product amplified from untreated macrophages or saline (control) tumor tissue samples were set as 1. A non-template control (NTC) was run in every assay, and all determinations were performed as triplicates for each animal in two separated experiments. The relative expression was calculated according to the equation Rel. Exp (RE) = 2^−ΔΔCt^. List of primers are detailed in the Table 1. The primer efficacy (PE) was calculated according to the equation PE (%) = {[10^(−1/m)^] − 1} × 100, m = slope and the PCR efficiency was shown by the LinRegPCR program^47^ Version 11.0.

**Table 1:** Specific forward and reverse primers.

	Forward	Reverse	Primer efficacy (%)	PCR efficiency (%)
TNF-α	5′-GACCCTCACACTCAGATCATCTTCT-3′	5′-CCACTTGGTGTTTTGCTACGA-3′	125.42	94.40
iNOS	5′-AAGATGGCCTGGAGGAATGC-3′	5′-TGCTGTGCTACAGTTCCGAG-3′	113.59	94.59
Arg1	5′-CAGAAGATTGGAAGAGTCAG-3′	5′-CAGATATGCAGGGAGTCACC-3′	105.68	94.80
IL-10	5′-GGTTGCCAAGCCTTATCGGA-3′	5′-ACCTGCTCCACTGCCTTGCT-3′	101.74	94.46
IL-1b	5′-TGACAGTGATGAGAATGACCTGTTC-3′	5′-TTGGAAGCAGCCCTTCATCT-3′	116.62	94.70
GAPDH	5′-CATCTCTGCCCCCTCTGCTG-3′	5′-GCCTGCTTCACCACCTTCTTG-3′	136.12	95.50

#### Isolation of intraperitoneal macrophages

Macrophages were isolated from the peritoneal cavity (pMϕ), incubated in serum-free medium for 30 min in a 24-well tissue-culture plate, and treated with 0.5 mM 4Mu or RPMI for additional 72 h. Then, cells were collected and used for flow cytometry analysis or maintained with Trizol (Invitrogen, USA) at − 80 °C. In addition, conditioned medium (CM) from 4Mu-treated pMϕ (4Mu-treated pMϕ derived-CM) or pMϕ derived-CM was stored at − 80 °C until its use. Flow cytometry of F4/80+, CD206+ and CD86+ were performed using a FACSAria (BD Biosciences, USA), macrophages type1/type2 proportion was calculated as above and quantitative PCR analysis were performed as we described above.

Hepa129 cells were incubated with RPMI (Hepa129 + RPMI), CM from isolated pMϕ (Hepa129 + pMϕ derived-CM) and conditioned media from isolated pMϕ in vitro treated with 4Mu (Hepa129 + 4Mu treated-pMϕ derived-CM) for 48 h. Preconditioned 1 × 10^6^ Hepa129 cells were used for in vivo experiments or for western blot. Intraperitoneal Mϕ also were obtained on day 9 and day 15 from mice with fibrosis-associated HCC treated with saline or 20 mg/kg 4Mu. Flow cytometry of F4/80+ CD86+ and CD206+ cells also were analyzed and the M1/M2 proportions were calculated as above.

#### Quantification of CD3+ cells on HCC tumors

Tumor lysates from mice treated with 4Mu or saline were obtained after liver perfusion with collagenase (Sigma-Aldrich, USA). Cell suspensions were then treated with RBC lysis buffer and stained with anti-CD4, anti-CD8, and anti-CD3 antibodies (BD Biosciences, USA). Then, lymphocytic infiltrate on tumor tissue was analyzed by flow cytometry.

#### Quantification of splenic CD11b^+^Gr1^+^ and CD4^+^ Foxp3^+^ cells from HCC-bearing mice

single cell suspensions of splenocytes were prepared, and resuspended in PBS (phosphate buffer solution). Cell suspensions were treated with red blood cell lysis buffer (0.15 M NH4Cl, 1 mM KHCO3, 0.1 mM Na2–EDTA), and washed with PBS 1% BSA. Splenocytes were stained with different conjugated-antibodies: anti Foxp3-PE (eBiosciences, USA), anti CD4-FITC, anti CD11b-APC, and anti-Gr1-PE (BD Biosciences, USA) and their respective isotypes. Then, cells were fixed with 1% paraformaldehyde and subjected to flow cytometry (Accuri 6C, BD, USA). Data were analyzed using Accuri 6C software.

#### Westen blotting

expression of TLR4, Sox2 and CD47 was detected in extracts from preconditioned whole, CD133+ or CD133- Hepa129 cells by immunobloting. Briefly, cells were collected and incubated in lysis buffer with protease inhibitors (50 mM Tris-HCI buffer, pH7.4, containing 0.1% Tween-20, 150 mM NaCl, 10 μg/ml aprotinin, 5 μg/ml leupeptin, 1 mM PMSF) 30 min on ice. Measurement of total protein concentration was performed using Bradford assay. Then, 50 μg of total protein was loaded and separated on 10% SDS-PAGE gels and transferred to polyvinylidene difluoride^48^. Blots were then developed with 1:500 mouse anti-TLR4 (Santa Cruz Biotechnology)^49^; 1:2000 rabbit anti-SOX2 (Santa Cruz Biotechnology)^50^ 1:1000 rat anti-CD47 (Abcam)^51^ and 1:5000 horseradish peroxidase (HRP)-conjugated goat anti-mouse antibody (Jackson ImmunoResearch Labs,USA)^52^. Bands were detected using the ECL detection system. Protein loading and transfer was monitored using an anti-actin antibody (1:1000, Santa Cruz Biotechnology, USA)^53^ and incubated with HRP-goat anti-mouse antibody (diluted 1/5000, Santa Cruz Biotechnology, USA)^52^. Bands intensities were measured by densitometer analysis using the Scion Image software (Scion Corporation, USA).

#### IL-6 quantification

To characterize conditioned medium, supernatants of pMϕ were collected 72 h after being treated with 4Mu, replaced with RPMI serum-free and collected after 24 and 48 h of culture. The concentration of IL-6 was measured by ELISA from BD (BD Biosciences, USA) according to the manufacture’s guideline.

#### Generation of bone marrow-derived DCs

DCs were generated from murine bone marrow cells as described previously^54^. Briefly, bone marrow of C3H/He mice or BALB/c were obtained from femurs and tibias, subjected to mechanic disruption, cell suspensions obtained and cultured with RPMI 1640 with 10% FBS, and IL-4 and GM-CSF (20 ng/ml; PeproTech, Germany). On day 3 and 5, the medium was withdrawn and replaced by new fresh RPMI. On day 7, suspension cells were collected (DCs) and used for experiments.

#### Phenotypic analysis of DCs

DCs were cultured with 0.5 mM 4Mu during 72 h. Then, DCs were stained with CD11c-PE, CD86-APC, and MCH-II-FITC (BD Biosciences, USA) and analyzed by flow cytometry.

#### Phagocytosis assay

DCs (5 × 10^4^) were immunostained with anti-CD11c, CD86 and MHCII and exposed to whole, CD133 + , CD133- Hepa129 cells treated or not with 4Mu (5 × 10^5^), labeled with DAPI and incubated for 2 h at 37 °C. Phagocytosis was determined by flow cytometry detection of CD11+ CD86+ MHCII+ DAPI+ cells.

#### Antigen loading

Hepa129, BNL cells or tumor extracts from HCC bearing-mice were obtain as previously^54^. Briefly, DCs were pulsed with cells or tumor lysates alone (200 μg/10^6^ cells/ml) at 37 °C for 18 h. Cells were then centrifuged, characterized by flow cytometry, and used for in vivo experiments.

### In vitro assays

#### Cell isolation by magnetic-activated cell sorting (MACS)

Hepa129 cells were labeled with primary CD133/1 antibody (Miltenyi Biotec, Germany), and the CD133 + was subsequently magnetically isolated using MACS Columns.

### Statistical analysis

All experiments were repeated at least 2 or 3 times on different occasions. Values were expressed as the mean ± SEM. Mann–Whitney, Tukey’s or Kruskall–Wallis (ANOVA) multiple comparison tests were used to evaluate the statistical differences between groups. Mice survival was analyzed by a Kaplan–Meier curve. *P* value < 0.05 was considered as significant. Prism software (Graph Pad, San Diego, CA, USA) was employed for the statistical analysis^25^.

## Supplementary Information

Supplementary Legends.

Supplementary Figures.
