# Supplementary material for: 4-methylumbelliferone-mediated polarization of M1 macrophages correlate with decreased hepatocellular carcinoma aggressiveness in mice
Source: Sci Rep. 2021 Mar 18;11:6310. doi: 10.1038/s41598-021-85491-0 (PMC7973733; doi:10.1038/s41598-021-85491-0)
Supplement: Supplementary file 1 — Supplementary Legends. [file 41598_2021_85491_MOESM1_ESM.docx]

**Supplementary Figure 1:** Representative images of Sirius Red stained-liver section from fibrotic mice treated with thioacetamide (TAA) during 4 weeks. Images were obtained with NIS-Elements Nikon microscope imaging proprietary software F3.00, SP7 Version 441.0308.3200.090723 (www.nikonmetrology.com)

**Supplementary Figure 2:** Myeloid derived-suppressor cells (MDSCs) and CD4^+^Foxp3^+^ regulatory T cells (Tregs) from spleen of saline or 4Mu treated HCC-bearing C3H mice were analyzed by flow cytometry using BD Accuri C6 Software Version 1.0.264.21 ([www.AccuriCytometers.com](http://www.AccuriCytometers.com)) A) Percentage of splenic Gr1+CD11b+ cells and B) splenic CD4+Foxp3+ cells were significantly reduced in 4Mu treated animals (n=4) compared to no treated mice (n=4). ***p<0.001 and *p<0.05 4Mu vs. saline, Mann-Whitney test. Data are expressed as mean percentage ± SEM from 2 independent experiments.

**Supplementary Figure 3:** DCs were cultured with 0.5 mM 4Mu for 72 h. The analysis of the percentage of CD11^+^MHCII^+^CD86^+^ was performed using flow cytometry *p<0.05 4Mu vs. RPMI, Mann-Whitney test. Data are expressed as mean percentage ± SEM from 3 independent experiments

**Supplementary Figure 4:** Full-length blots from Figure 3 B and C. A) TLR4, CD47 and Sox2 expression of Hepa129 cells 24 or 48h cultured with pMφ-derived CM or 4Mu-treated pMφ-derived CM. B) TLR4, CD47 and Sox2 expression was also determined by western blot on magnetic-isolated CD133^+^ and CD133^-^ Hepa129 cells 24 or 48 h cultured with pMφ-derived CM and 4Mu-treated pMφ-derived-CM.
