# Supplementary material for: 4-methylumbelliferone-mediated polarization of M1 macrophages correlate with decreased hepatocellular carcinoma aggressiveness in mice
Source: Sci Rep. 2021 Mar 18;11:6310. doi: 10.1038/s41598-021-85491-0 (PMC7973733; doi:10.1038/s41598-021-85491-0)
Supplement: Supplementary file 2 — Supplementary Figures. [file 41598_2021_85491_MOESM2_ESM.pdf]

4-methylumbelliferone-mediated polarization of M1 macrophages correlate with decreased hepatocellular carcinoma aggressiveness in mice

Marcelo M. Rodríguez[1], Agostina Onorato[1], María José Cantero[1] , Luciana Domínguez[1], Juan Bayo[1], Esteban Fiore[1], Mariana García[1] , Catalina Atorrasagasti[1], Ali Canbay[2], Mariana Malvicini[1*], Guillermo D. Mazzolini [1,3*]

[1]Gene Therapy Laboratory, Instituto de Investigaciones en Medicina Traslacional, Facultad de Ciencias Biomédicas, CONICET- Universidad Austral, Buenos Aires, Argentina.

[2]Department of Medicine, Universitätsklinikum Knappschaftskrankenhaus Bochum, Ruhr-Universität Bochum, Germany

[3]Liver Unit, Hospital Universitario Austral, Universidad Austral, Buenos Aires, Argentina

*Shared credits for senior authorship

Supplementary Figure 1

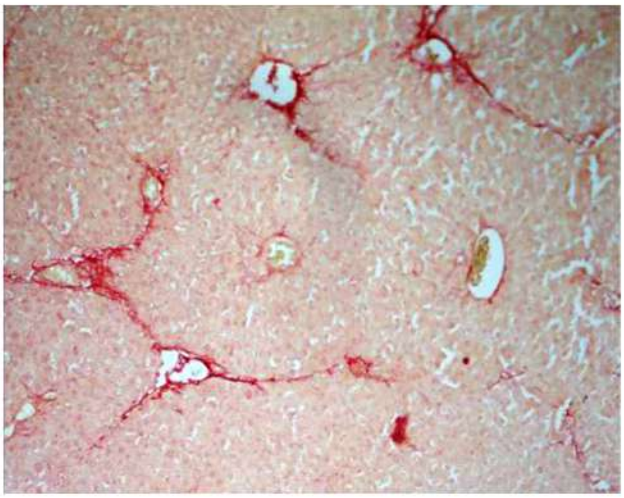

Supplementary Figure 2

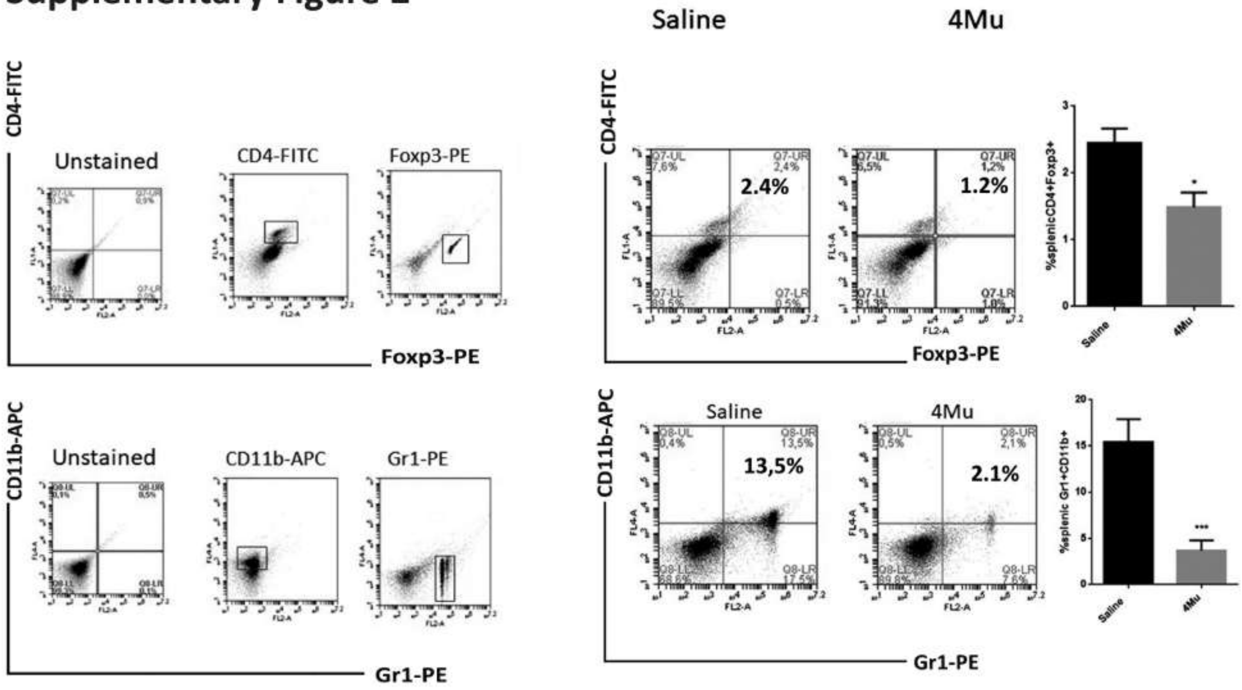

Supplementary Figure 3

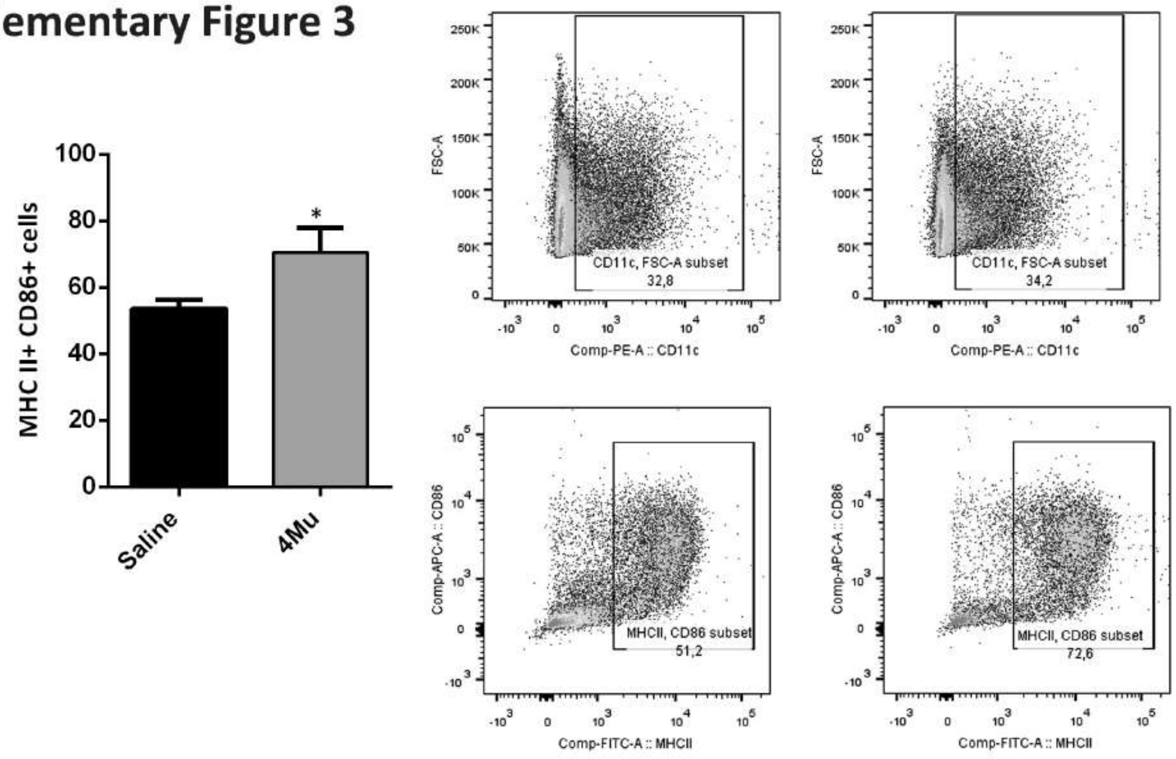

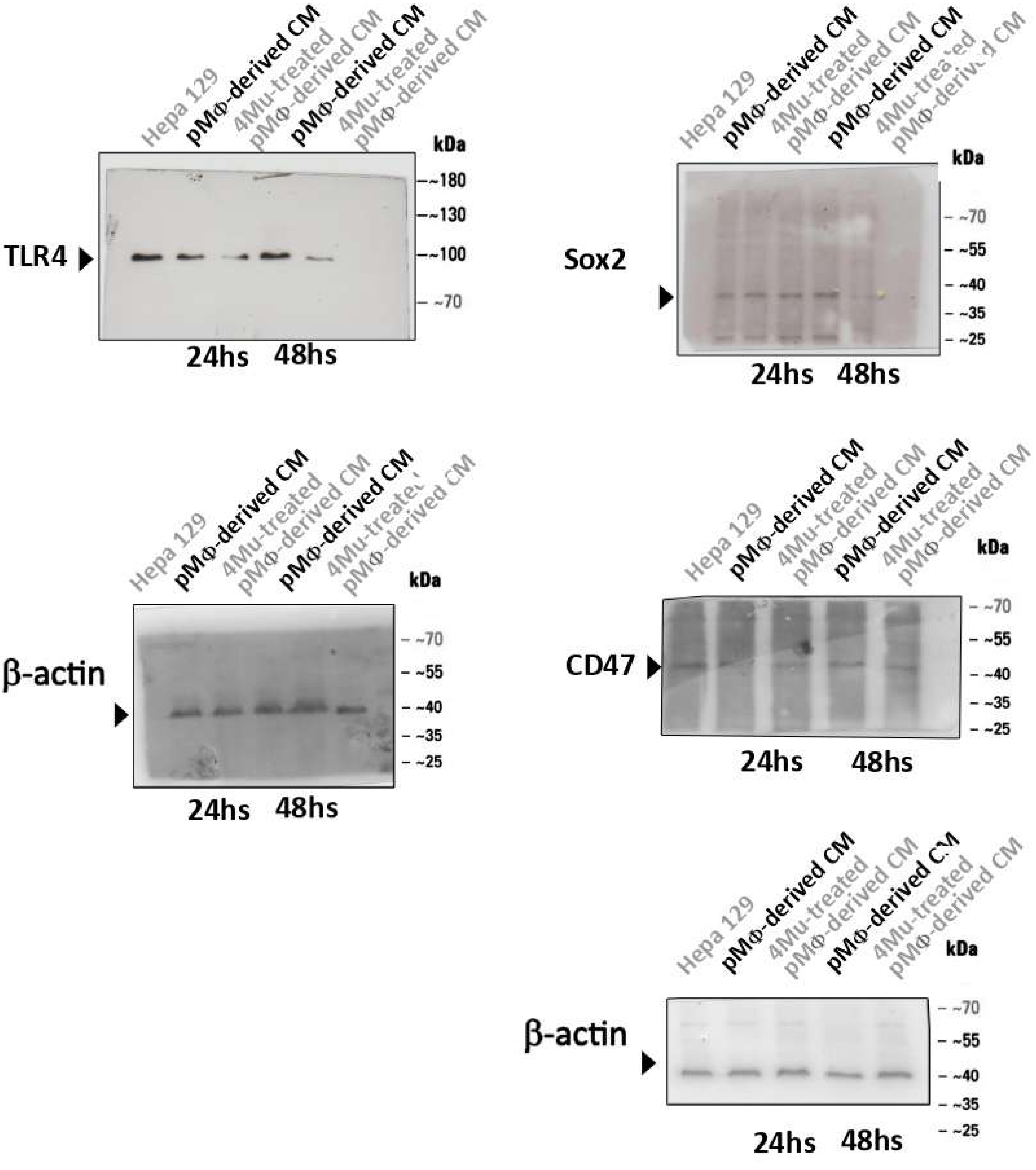

supplementary figure 4A

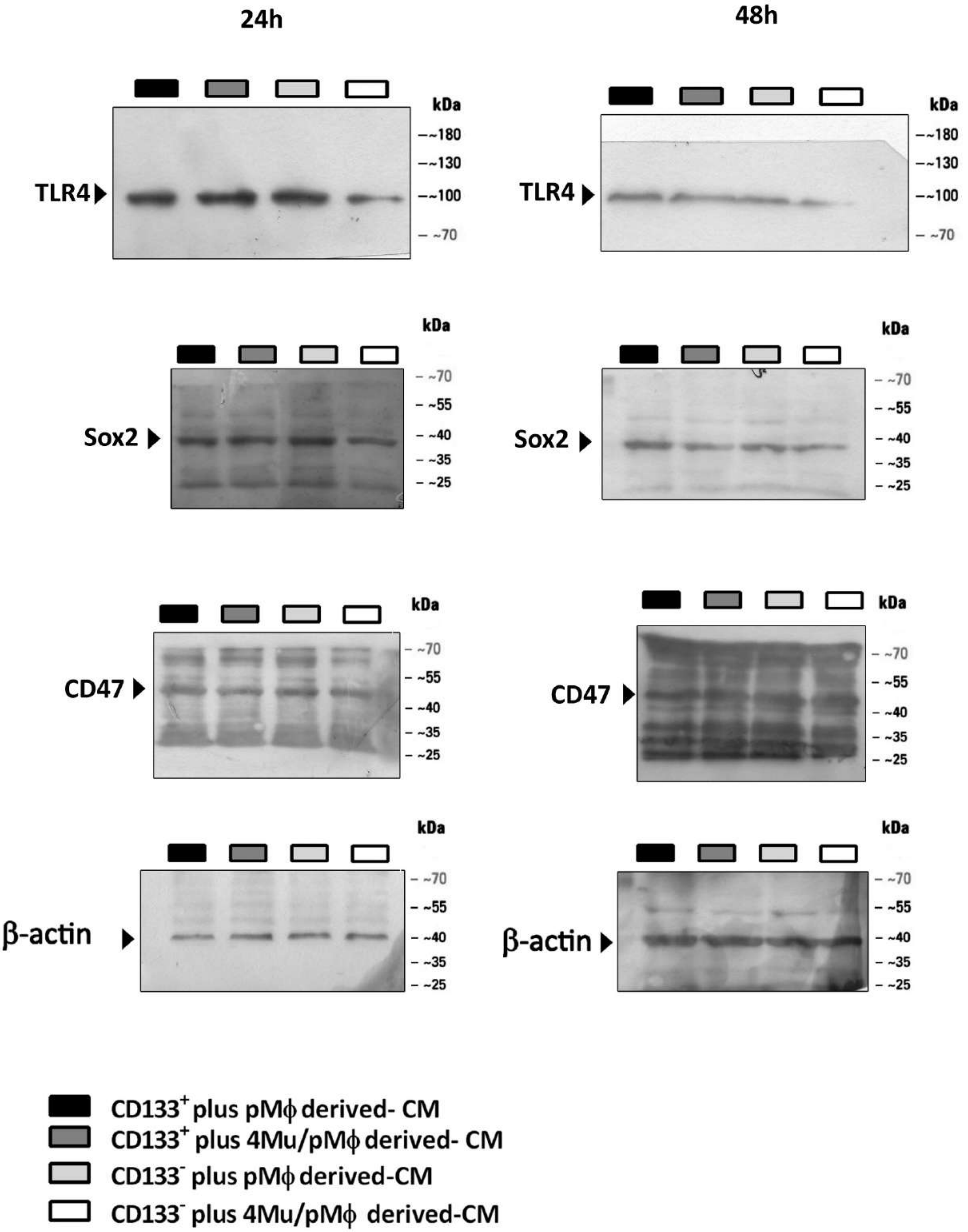

supplementary figure 4B
